# SLKIR: A framework for extracting key information from air traffic control instructions Using small sample learning

**DOI:** 10.1038/s41598-024-60675-6

**Published:** 2024-04-29

**Authors:** Peiyuan Jiang, Chen Zeng, Weijun Pan, Boyuan Han, Jian Zhang

**Affiliations:** 1 Air Traffic Management College, Civil Aviation Flight University of China, Guanghan, 618307 China; 2 Xinjin Branch, Civil Aviation Flight University of China, Chengdu, 611431 China

**Keywords:** Computer science, Scientific data, Engineering, Aerospace engineering

## Abstract

In air traffic control (ATC), Key Information Recognition (KIR) of ATC instructions plays a pivotal role in automation. The field's specialized nature has led to a scarcity of related research and a gap with the industry's cutting-edge developments. Addressing this, an innovative end-to-end deep learning framework, Small Sample Learning for Key Information Recognition (SLKIR), is introduced for enhancing KIR in ATC instructions. SLKIR incorporates a novel Multi-Head Local Lexical Association Attention (MHLA) mechanism, specifically designed to enhance accuracy in identifying boundary words of key information by capturing their latent representations. Furthermore, the framework includes a task focused on prompt, aiming to bolster the semantic comprehension of ATC instructions within the core network. To overcome the challenges posed by category imbalance in boundary word and prompt discrimination tasks, tailored loss function optimization strategies are implemented, effectively expediting the learning process and boosting recognition accuracy. The framework's efficacy and adaptability are demonstrated through experiments on two distinct ATC instruction datasets. Notably, SLKIR outperforms the leading baseline model, W2NER, achieving a 3.65% increase in F1 score on the commercial flight dataset and a 12.8% increase on the training flight dataset. This study is the first of its kind to apply small-sample learning in KIR for ATC and the source code of SLKIR will be available at: https://github.com/PANPANKK/ATC_KIR.

## Introduction

### Background

**Figure 1:**
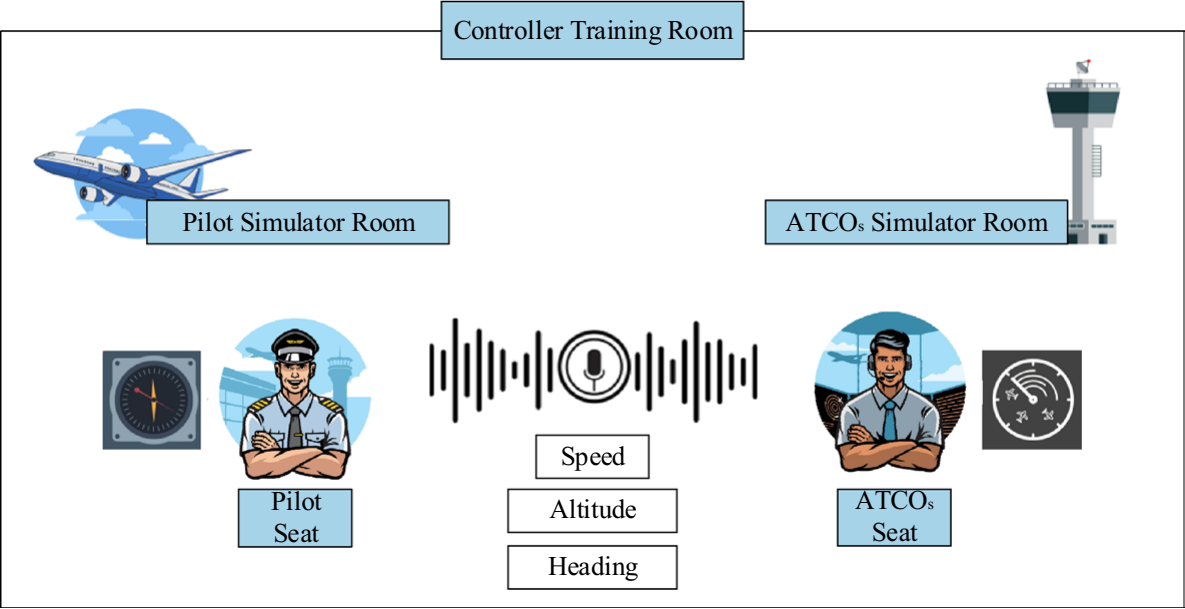
Controller Training Scenario Diagram.

**Figure 2:**
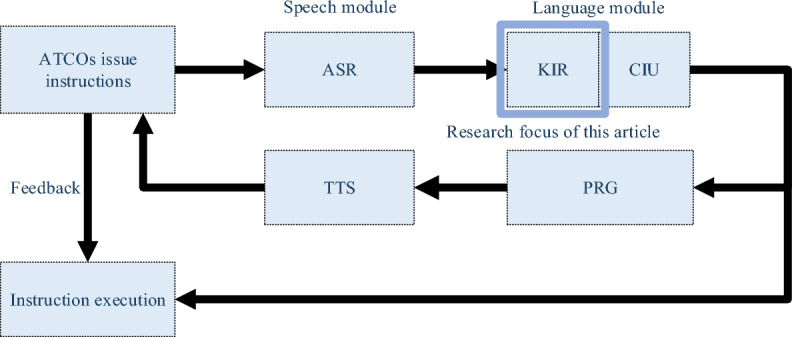
Intelligent Pilot Technology Process Diagram.

The swift advancements in deep learning have significantly accelerated the path to automation in a broad spectrum of application areas. For example, in the transportation domain, Emin Güney and his team at Sakarya University of Applied Sciences have made notable strides with the creation of a real-time Advanced Driver-Assistance System. This system, characterized by its low energy consumption and the integration of a high-speed mobile GPU platform, leverages deep learning to enable robust detection of traffic signs and targets^1^. Moreover, this group has ventured into the realm of automated charging for maritime vessels, culminating in the development of an innovative autonomous dockside robotic charging system for ships^2,3^, showcasing the immense potential of deep learning technologies in the advancement of automation. In the field of Air Traffic Control (ATC), automated pilot agent systems represent a significant area of research. Air Traffic Control Officers (ATCOs) must complete simulated equipment training before obtaining qualifications to work in real ATC scenarios^4,5^. The training scenarios are depicted in Fig. 1. The control simulator training room includes both a pilot seat room and a controller seat room, as shown in Fig. 1. This means that a control trainee cannot complete a training session alone; they also need a companion to operate the pilot seat. Additionally, AI can be used to replace the pilot seat, thereby reducing training costs and enhancing the efficiency of controller training^6,7^. The core technologies used to replace manual pilot seats include: Automatic Speech Recognition (ASR), Key Information Recognition (KIR), Controlling Instruction Understanding (CIU), Pilot Repetition Generation (PRG), Text to Speech (TTS), etc.^8^. The technical process flow is illustrated in Fig. 2.

KIR is used to transform unstructured text outputs from ASR into structured texts that are understandable by machines. In automated pilot agent systems, the importance of KIR technology is akin to the role of object detection technology in autonomous driving systems^9^. As of now, some experts and scholars in the field of ATC have conducted research on related technologies. For example, Zhang J, Pan W have extensively explored PRG technology within intelligent pilot systems, highlighting the crucial role of KIR technology^10,11^. Lin Yi's team constructed a multi-task shared encoder network based on Bidirectional Long Short-Term Memory (BiLSTM) and Multi-Layer Perceptron (MLP). This network effectively converts ATC voice instructions into machine-understandable control intentions and instruction parameters ^12^. Zuluaga-Gomez J and others built an advanced entity parsing system in the intelligent pilot architecture by fine-tuning pre-trained language models (LM)^13^ and achieved commendable results on the ATCO2 dataset^14^.

### Motivation

Despite these studies advancing KIR technology, two key issues regarding KIR in the ATC field still need further breakthroughs. The first breakthrough needed is addressing the issue of insufficient model performance on small sample data. Obtaining data in the ATC field is extremely difficult due to data confidentiality. Moreover, the obtained raw ATC data must be labeled by professionals before it can be used, and the cost of annotation is high^15^. The performance of data-driven models critically depends on the quantity and quality of data. Therefore, it is crucial to research how to achieve efficient KIR of ATC on small sample data. The second breakthrough needed is addressing the issue of poor generalizability of models to unseen samples. Generalizability is a core assessment metric for data-driven models^16^. ATC commands vary significantly in vocabulary across different types and locations. For instance, in commercial flights, terms like 'pushback', 'taxi', and 'takeoff' are only applicable within the scope of airport terminal areas; similarly, the waypoint "EKOKA" is used only in specific areas, namely the Chengdu region of China^17^. In training flights, the call signs of training aircraft differ significantly from those of commercial aircraft.

### Contributions

This paper employs transfer learning and efficient model construction techniques to develop a robust KIR model for ATC driven by small sample data. Specifically, our contributions include:

- We conducted an in-depth study on constructing a robust KIR model driven by small sample data. Specifically, we built an end-to-end KIR deep learning framework based on the MHLA mechanism.
- We cleverly designed a discriminative task based on prompt information. By discriminating whether the control instructions contain externally inputted prompt information, we enhance the semantic understanding of the input control instructions by the backbone network.
- We proposed a loss function optimization strategy for addressing the issue of boundary word information sparsity in the KIR process. This optimization strategy can enhance the model’s learning capability.
- Building on previous research^10,11^, and considering the characteristics of air traffic control as well as the key information required by intelligent agents to execute instructions, this paper provides a detailed classification of key information categories in control instructions, including Callsign, Action, Action Value, and Condition.

## Related Work

**Figure 3:**
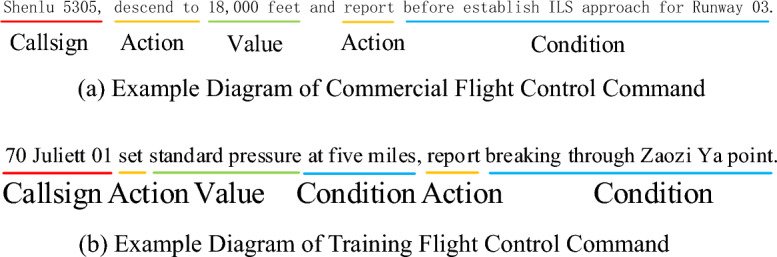
Key Information Categories of ATC Instructions.

KIR of ATC can be classified as a Named Entity Recognition (NER) task in natural language processing (NLP). Currently, research in NER has evolved from the initial flat NER^18^ to overlapping NER^19^, and further developed into discontinuous NER^20^. Flat NER refers to entities in the data that do not overlap with each other and have a simple structure, such as names of people or places, with each entity being tagged as a continuous, non-nested category. Overlapping NER refers to the presence of overlapping entities in the data, where a segment of an entity can belong to multiple entity categories simultaneously, or one entity may contain another entity within it. Discontinuous NER refers to entities in the data that are composed of non-continuous text segments but together represent a semantically complete entity. The specific type of NER researched depends on the characteristics of the dataset. ATC instructions are characterized by being concise and unambiguous. Therefore, KIR of ATC can be further categorized into the flat NER category. In Fig. 3, the key information categories of ATC instructions are illustrated. Figure 3a shows ATC instructions for commercial flights, while Fig. 3b shows ATC instructions for training flights.

In flat NER problems, sequence labeling and span-based methods are the two main solutions. Sequence labeling achieves NER by assigning a label category to each character. Selim F. Yilmaz and his team employed sequence labeling, building a Bidirectional Long Short-Term Memory (BiLSTM)- Conditional Random Field (CRF) model for NER tasks. This model can effectively handle noisy texts across multiple domains^21^. Souza F proposed a BERT-CRF model^22^, and the experimental results showed that pre-trained language models can significantly enhance model performance on small sample datasets. Diao S used a pre-trained model as an encoder and incorporated N-gram combinations of different characters as additional information into the model to enhance entity boundary discrimination, achieving state-of-the-art (SOTA) performance on multiple Chinese datasets^23^. Zhu W enhanced the performance of the model by incorporating input character type information as supplementary data into BERT^24^. Similarly, Liu W and his team introduced external lexical information into the underlying encoding process of BERT, achieving state-of-the-art (SOTA) results on multiple Chinese NER datasets^25^. J Chu and colleagues proposed a multi-feature fusion Transformer architecture that significantly enhances the model’s performance by augmenting sentence semantic information through the fusion of features such as Chinese characters and radicals^26^. However, sequence-based labeling tasks present several challenges. These include: 1. Labeling category tags for each character is a time-consuming and labor-intensive process. 2. The introduction of external dictionaries leads to additional consumption of computational resources. Span-based methods are a more direct and effective novel approach where entity recognition is viewed as a span classification task^27^. Shen Y and his team viewed NER as a joint task of boundary regression and span classification, proposing a two-stage method—first localization then marking—to achieve efficient NER^19^. Li J and others pointed out that simulating the adjacency relationships between entity words is key to achieving unified NER. Based on this, they proposed a model called W2NER, which unifies the handling of flat, overlapping, and discontinuous entities. This model achieved state-of-the-art (SOTA) results on multiple Chinese datasets^28^. Lu Y and his team proposed a universal information extraction model suitable for small sample data. This model has achieved leading results in Chinese entity extraction, relation extraction, and other information extraction tasks^29^. We have summarized the related work in this section and presented it in Table 1, to facilitate the reader’s further understanding of the details of the aforementioned literature. The columns of the table represent the reference number, the dataset used, the language types involved in the dataset, the entity recognition method employed, the model techniques it is based on, and the availability of the data. Note that if no information is available in the reference paper, we set it as ‘No’. In general fields, although research methods for flat NER continue to advance and develop, when these advanced methods are applied to the ATC domain, their performance generally significantly decreases. This is mainly due to the significant linguistic style differences between ATC instructions and standard Mandarin. For example, in ATC instructions, numbers frequently appear in various information such as aircraft call signs, flight altitudes, atmospheric pressure, speed, headings, runway numbers, etc., as shown in Fig. 3, which greatly interferes with the determination of key information categories.

**Table 1:** Literature Summary Table of Related Work.

Reference	Dataset	Language	Method	Technology	Availability
^18^	CoNLL02 CoNLL03	Spanish Dutch English German	Sequence labeling	LSTM-based	Publicly available
^19^	ACE04 ACE05 KBP17 GENIA	English	Span-based	BiLSTM-based	Publicly available
^20^	CLEF-Dis CADEC GENIA ACE05	English	Span-based	BiLSTM-based	Publicly available
^21^	Turkish NER	Turkish	Sequence labeling	BiLSTM-based	No
^22^	HAREM I	Portuguese	Sequence labeling	BERT-based	Publicly available
^23^	MSRA	Chinese	Sequence labeling	BERT-based	Publicly available
^24^	No	Chinese	Sequence labeling	BERT-based	No
^25^	Weibo Ontonotes MSRA Resume	Chinese	Sequence labeling	BERT-based	Publicly available
^26^	Aerospace dataset	Chinese	Sequence labeling	Transformer- based	No
^27^	ACE04 ACE05 SciERC WLPC	English	Span-based	BiLSTM-based	Publicly available
^28^	CoNLL03 OntoNotes MSRA Weibo Resume ACE04 ACE05 GENIA CADEC ShARel	English Chinese	Span-based	BERT-based	Publicly available
^29^	ACE04 CoNLL03	English German	Span-based	ERNIE-based	Publicly available

In this study, a robust deep learning framework for KIR of ATC driven by small sample data is proposed. The framework can effectively improve recognition accuracy without the need for external ATC dictionaries, and demonstrates strong generalizability on small sample datasets. The structure of the remainder of this paper is as follows: "SLKIR Framework" provides an overview of the proposed framework, detailing its main components. "Experiments" delves into the experimental datasets, the environment, and the results, offering a comprehensive discussion. Finally, "Conclusion" concludes the paper and proposes directions for future research.

## SLKIR framework

**Figure 4:**
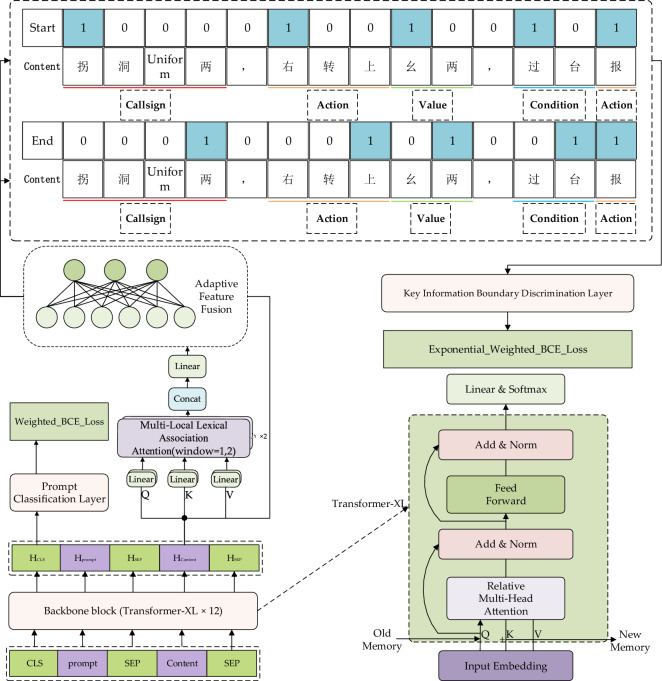
SLKIR Framework Diagram.

The SLKIR primarily consists of three components: the backbone network, a prompt classification layer, and a key information boundary word discrimination layer, with its framework illustrated in Fig. 4. The backbone network employs 12 Transformer-XL encoding blocks, whose pre-trained parameters are derived from the Chinese pre-trained language model Ernie3.0^30^. For pre-trained language models, prompts^31–33^ are crucial in guiding the generative direction of the model. Furthermore, existing studies have shown that multi-task learning strategies are helpful in enhancing the generalization of models^34^. Thus, a prompt classification layer was added to enhance the semantic expressive capacity of the backbone network. The key information boundary word discrimination layer comprises two linear layers, each dedicated to identifying the start and end boundaries of key information. Compared to using a single linear layer, using two linear layers allows the model to learn faster and achieve higher performance. Given that the key information boundary words in ATC instructions are closely linked to characters within a narrow surrounding context, a MHLA mechanism is utilized to capture the latent representations associated with these boundary words. These latent representations are integrated into the outputs of the backbone network through an adaptive fusion module to improve the accuracy of boundary word discrimination. Due to the fact that the actual number of boundary words, which are crucial in input sentences, is significantly smaller than the number of non-boundary words, and considering the class imbalance in positive and negative samples in the prompt classification task, Exponentially Weighted BCE Loss (hereinafter referred to as EBCE) and Weighted BCE Loss (WBCE) were employed to further optimize the model training process and enhance the model's learning capability.

### Backbone block

The Transformer-XL block in the backbone network is a variant of the Transformer^35^ that introduces a segment-level recurrence mechanism. This modification enables it to effectively learn and process longer text content. Furthermore, the pre-trained parameters of the Transformer-XL block come from Ernie3.0. Ernie3.0 integrates a substantial amount of knowledge graph data during its training process. This endows the Transformer-XL block with robust zero-shot and few-shot learning capabilities, making it suitable for handling various natural language understanding and generation tasks. For an input sequence of length n, *X* = {*x*_*1*_*, x*_*2*_*, …, x*_*n*_}, the backbone network transforms it into high-dimensional word vectors containing contextual information, denoted as *H* = {*h*_*1*_*, h*_*2*_*, …, h*_*n*_}, where *H* ∈ *R*^*n*×*dh*^ and d_h_ represents the dimension of the word vectors. Our input format is CLS/prompt/SEP/Content/SEP, due to the incorporation of external prompt information. The relationship between prompt and Content is illustrated in the following examples: CLS/Callsign/SEP/Shunfeng 5137, maintain 7500/SEP; CLS/Condition/SEP/Shunfeng 5137, maintain 7500/SEP. In the two cases above, the prompt in the former exists within the Content, while the prompt in the latter does not exist within the Content. This is the basis for constructing a prompt classification task to deepen the model's semantic understanding of the Content. Furthermore, the CLS token indicates the beginning of the input sequence, and its high-dimensional word vector is used as a comprehensive semantic representation of the entire input sequence. The SEP token is used to separate the prompt and the content, and to mark the end of the input sequence. The use of the SEP token is effective for the model to understand different parts of the input data, thereby facilitating more efficient learning and prediction.

### Multi-head local lexical association attention and adaptive fusion module

**Figure 5:**
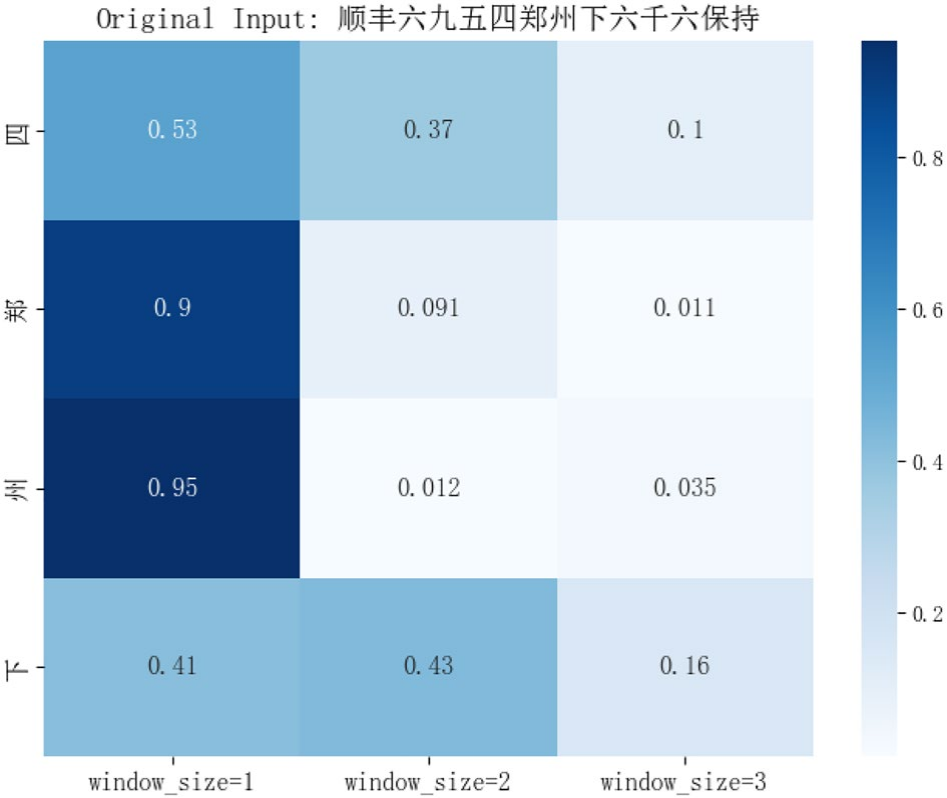
Example Diagram of Correlation between Key Information Boundary Words and Characters at Different Distances.

We analyzed 10,000 collected, unlabelled transportation flight text data using statistical methods. Specifically, we calculated the co-occurrence frequency of each boundary word with characters at distances of 1, 2, and 3 (measured in characters) to analyze the association of key information boundary words with characters at these varying distances. It was found that the boundary of key information has a strong correlation with nearby characters and a weaker correlation with characters farther away. Figure 5 presents a visualization of the data analysis. Based on this discovery, a MHLA mechanism has been proposed to increase the mutual information of boundary words, thereby enhancing the discrimination accuracy of these boundary words. Specifically, for the input sequence *X* = {*x*_*1*_*, x*_*2*_*, …, x*_*n*_}, the computation process of the MHLA is as follows.

#### Initialization transformation

For the given query vector *Q*, key vector *K*, and value vector *V*, where *Q* ∈ *R*^*dq*^, *K* ∈ *R*^*dk*^, *V* ∈ *R*^*dv*^, and *d*_*q*_ = *d*_*k*_ = *d*_*v*_, three sets of linear transformations are used to map these vectors into a new feature space. In this space, the latent representations related to key information boundary words can be more effectively captured by the model.

1 $$ Q = W^{Q} \cdot X + b^{Q} $$

2 $$ K = W^{K} \cdot X + b^{K} $$

3 $$ V = W^{V} \cdot X + b^{V} $$

In Eqs. (1–3), *W*^*Q*^, *W*^*K*^ and *W*^*V*^ are three learnable weight matrices, while *b*^*Q*^, *b*^*K*^, and *b*^*V*^ are the corresponding bias terms.

#### Local attention calculation

(1) For the *i*-th character in the sequence, its local attention span is [*max*(0, *i-window_size*), *min*(*window_size*, *i* + *window_size* + 1)], where *window_size* is the distance from the character *i* to the furthest element that needs attention, centered around character *i*.

(2) Attention Score and Probability Calculation

4 $$ attn\_scores_{i} = \frac{{QK_{i}^{T} }}{{\sqrt {hidden\_size} }} $$

5 $$ attn\_probs_{i} = \frac{{e^{{attn\_scores_{i} }} }}{{\sum\nolimits_{j = 1}^{n} {e^{{attn\_scores_{j} }} } }} $$

In Eqs. (4–5), *hidden_size* is the dimension of vector *K*, *attn_scores*_*i*_ represents the attention score of query vector *Q* with respect to the *i*-th key vector *K*; *attn_probs*_*i*_ is the attention probability of query vector *Q* with respect to the *i*-th key vector *K*.

(3) Attention Output

6 $$ attn\_output = \sum\limits_{i = 1}^{n} {attn\_probs_{i} } \, \cdot \, V_{i} $$

#### Multi-head initialization

Before computing MHLA, the number of attention heads and the attention span for each head should be predetermined.

#### Multi-head attention calculation

(1) Assuming head_j_is the j-th attention head, its computation process is as follows.

7 $$ output_{j} = LocalAttention(X,window\_size) $$

In Eq. (7), the process of *LocalAttention*() function is as shown in step (2), and *output*_*j*_ is the output of the *j*-th attention head.

(2) Head output combination

The final attention result is calculated as the average sum of the outputs from all attention heads, as follows.

8 $$ Combined\_output = \frac{1}{num\_heads}\sum\nolimits_{j = 1}^{num\_heads} {Output_{j} } $$

In Eq. (8), *Combined_output* represents the output result of the MHLA, and *num_heads* is the total number of heads in the MHLA mechanism. In this study, *num_heads* was set to 2, and *window_size* was set to 1 and 2 respectively.

#### Adaptive fusion module

The adaptive fusion module is implemented based on linear transformations. It is used to fuse the features outputted by the MHLA with the features outputted by the backbone network, updating the parameters required for fusion through backpropagation during the training process, in order to learn the importance of different features for the final task. For the output sequence *H* = {*h*_*1*_*, h*_*2*_*, …, h*_*n*_} of the backbone network and the output sequence *F* = {*f*_*1*_*, f*_*2*_*, …, f*_*n*_} of MHLA, the fusion formula is as follows.

9 $$ C = concat(H,F,dim = - 1) $$

10 $$ O = W \cdot C + b $$

In Eqs. (9–10), *C* is the result of concatenating the corresponding elements of the output sequences *H* and *F* along the last feature dimension. The *Concat* function is used to implement the concatenation of vectors. *O* is the output of the adaptive module, *W* is the weight matrix of the linear layer, and *b* is the bias vector.

### Loss function

#### EBCE loss

The EBCE loss function is proposed for binary classification problems where positive and negative samples are severely imbalanced and the ratio is uncertain. It applies exponential weights to sparse sample categories on the basis of BCE loss, enabling the model to pay more attention to these categories during training. Its specific calculation formula is as follows.

(1) When calculating the loss of the model on a single sample, an exponential weight is assigned to the BCE loss value of each element in the sample. The formula for calculating the weights is as follows.

11 $$ w_{i} = e^{{(a.y_{i} )}} $$

In Eq. 11, *w*_*i*_ is the weight corresponding to the BCE loss value of the *i*-th element, *y*_*i*_ is the label value (0 or 1) corresponding to the *i*-th element, and *α* is a scaling factor used to regulate the weight of the loss value for a certain category, which is set to 2 in this paper.

(2) The formula for calculating the weighted loss value of *i*-th element is as follows.

12 $$ WeightBCE_{{\text{i}}} = BCE_{i} \cdot w_{{\text{i}}} $$

(3) The final calculation formula for the EBCE loss of a sample is as follows.

13 $$ EBCELoss = \frac{1}{m}\sum\nolimits_{{{\text{i}} = 1}}^{m} {WeightBCE_{i} } $$

In Eq. (13), m represents the number of elements in the sample.

#### WBCE loss

(1) The WBCE loss is designed for situations where the data category imbalance ratio is known. It allocates a proportionate weight to the loss value of samples in different categories based on the BCE loss. The difference between WBCE and EBCE lies in the calculation of weights. The formula for calculating the weights for WBCE is as follows.

14 $$ w_{i} = (1 - y_{i} )weight\_zero + y_{i} weight\_one $$

In Formula (14), when *y*_*i*_ equals 1 (positive class), *w*_*i*_ is equal to *weight_one*; when *y*_*i*_ equals 0 (negative class), *w*_*i*_ is equal to *weight_zero*. In this paper, based on the ratio of positive to negative classes, *weight_zero* and *weight_one* are set to 1.68 and 0.71, respectively.

(2) The final WBCE loss calculation formula is as follows, where the calculation process of *weightBCE*_*i*_ is shown in Eq. (12).

15 $$ WBCELoss = \frac{1}{m}\sum\nolimits_{{{\text{i}} = 1}}^{m} {WeightBCE_{i} } $$

#### Final Loss Function

16 $$ Loss = \frac{1}{3}\left( {start\_EBCELoss + end\_EBCELoss + WBCELoss} \right) $$

In Eq. (16), *start_EBCELoss* refers to the loss generated in identifying the start boundary words of key information, *end_EBCELoss* refers to the loss generated in identifying the end boundary words of key information, and *WBCELoss* refers to the loss generated in identifying the relationship between prompt and content.

## Experiments

### Dataset

**Figure 6:**
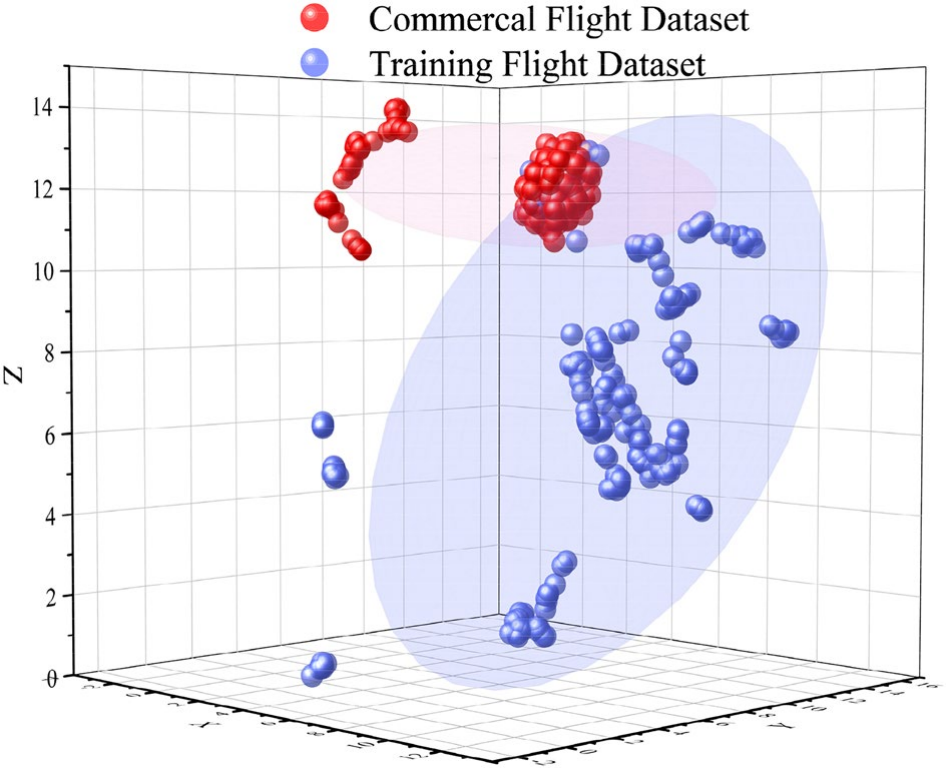
Three-Dimensional Visualization of Dataset Distribution Differences.

The ATC instruction data used consists of commercial flight ATC instruction datasets and training flight ATC instruction datasets. The commercial flight ATC instruction data is sourced from the voice transcription texts of the terminal area of an airport in East China, totaling 1204 records, with 1004 for model training and 200 for testing model performance. Training flight ATC instruction data was collected during the flight training sessions at the Civil Aviation Flight University of China. Within the gathered training flight ATC instruction dataset, 200 records have been annotated, specifically for testing the model's generalization capabilities. UMAP (Uniform Manifold Approximation and Projection), a nonlinear dimensionality reduction algorithm^36^, is used to extract features from the dataset, thereby enabling the visualization of the dataset distribution. Specifically, we first segmented the ATC instruction texts in the two datasets using the Jieba segmentation technology integrated with an ATC lexicon. Then, we applied the TF-IDF vectorization method to convert each line of text from the two datasets into a high-dimensional vector, where the dimensionality equals the number of unique segmented terms in the datasets. Finally, the TF-IDF vectors from these two datasets were used as input features for the UMAP algorithm, to visualize the distribution of the two datasets in a three-dimensional space. The results are shown in Fig. 6. Data annotation was carried out using the open-source tool doccano, with the annotator being licensed ATCO. The 'Aeronautical Radio Telephony Phraseology Standards (MH/T4014-2003)' published by the Civil Aviation Administration of China is used as a reference for data annotation. In this study, we categorize the key information in ATC instructions into four types from the perspective of the information required by agents to execute instructions: Callsign, Action, Action Value, and Condition. Previous works^10,11^ have highlighted the importance of Callsign, Intention, Action, and Parameters in control instructions. However, intention does not belong to the key information in the text, and the category of parameters is not specific enough. Based on this, we further divide parameters into two categories: Action Value and Condition. Furthermore, to enhance the model's ability to semantically understand control instruction information, we introduced a task to judge whether Content contains a prompt to promote the backbone network's semantic understanding of Content. As illustrated by the following two examples, CLS/Callsign/SEP/Shunfeng 5137, maintain 7500/SEP; CLS/Condition/SEP/Shunfeng 5137, maintain 7500/SEP. In these two examples, the prompts are respectively Callsign and Condition, with Content being Shunfeng 5137, maintain 7500. In these examples, the former is a positive example, meaning the Content contains Callsign information, while the latter is a negative example, indicating the Content does not contain Condition information. The negative samples for this task were constructed by identifying the key information categories not contained in each ATC instruction. Due to the limited number of ATC instructions lacking information categories, the number of negative samples was fewer than positive samples. Specifically, 1004 positive samples and 426 negative samples were constructed.

From Fig. 6, it can be observed that the commercial flight dataset is more tightly distributed in feature space, while the training flight dataset is more loosely distributed and exhibits significant differences from the commercial flight dataset, which can be used for generalizability validation.

### Experimental parameter configuration

SLKIR is implemented using the Pytorch framework. The configuration of our training server is detailed as follows: it operates on Windows 10, utilizes an E5-2680 V4 @ 2.40GHZ CPU, and is equipped with an RTX 2080Ti GPU. The hyperparameter settings for SLKIR are outlined in Table 2.

**Table 2:** SLKIR Hyperparameters.

Hyperparameter	Value
Hidden_size	768
Num_heads	2
Window_size	1&2
Scale_factor	2
Cls_weight	1.68&0.71
Max sequence length	60
Optimizer	Adam
Learning rate	0.0001
Batch size	64
Number of epoch	200
Patience	10

In Table 1, hidden_size refers to the feature dimension of each character output by the Transformer-XL block; num_heads denotes the number of local vocabulary-associated attention heads; window_size is the radius of attention; scale_factor is the scaling factor in EBCEloss; cls_weight represents the weight of each category in WBCEloss, inversely proportional to the frequency of that category; max sequence length is the maximum length of the input information. The optimizer used is Adam, with a learning rate of 0.0001. The model is trained in batches of 64, for a total of 200 training epochs. Additionally, an early stopping strategy based on validation loss is implemented during training. A 'patience' value of 10 indicates that training will stop if the validation loss does not decrease below the previous best loss for 10 consecutive training epochs. The experimental evaluation metrics use the micro-averaging approach for precision, recall, and F1 score^37^, with the formulas for these metrics provided as follows.

17 $$ P = \frac{TP}{{TP + FP}} $$

18 $$ R = \frac{TP}{{TP + FN}} $$

19 $$ F1 = \frac{2PR}{{P + R}} $$

In Eqs. (17–19), P represents precision, R represents recall, F1 represents the F1 score; TP represents true positives predicted by the model, TN represents true negatives predicted by the model, FP represents false positives predicted by the model, and FN represents false negatives predicted by the model.

### Ablation study

#### Validation of strategy effectiveness

In this section, ablation studies were conducted to verify the effectiveness of MHLA and the multi-task strategy, with the experimental results shown in Table 3. In Table 3, Ernie3.0-Softmax is used for Baseline, while for Baseline-MHLA, the Ernie3.0-Softmax model is enhanced with MHLA, where num_heads is set to 2 and window_size is set to 1 & 2. The Ernie3.0-Softmax model, which incorporates the prompt-based discriminative task, is represented by Baseline-CLS_task. Meanwhile, Baseline-MHLA-CLS_task signifies the integration of both the MHLA mechanism and the prompt-based discriminative task into the Ernie3.0-Softmax model. The datasets selected for testing include the test sets of the commercial flight ATC instruction dataset and the training flight ATC instruction dataset, with the evaluation metric being the F1 score.

**Table 3:** Results of Model Performance Impact by Different Strategies.

Experiment number	Model name	Commercial flight data	Training flight data
1	Baseline	0.838	0.729
2	Baseline-MHLA	0.850	0.749
3	Baseline-CLS_task	0.841	0.758
4	Baseline-MHLA-CLS_task	**0.851**	**0.790**

Significant values are in bold.

Experiment 1 presents the test results of the baseline model, while Experiment 2 shows the results of the baseline model after adding the MHLA module. Comparing Experiment 2 with Experiment 1, it can be seen that the addition of the MHLA module resulted in a relative improvement of 1.43% in performance on the commercial flight dataset and a 2.74% improvement on the training flight dataset compared to the baseline model. Experiment 3 displays the test results of the baseline model with the introduction of the CLS_task, and Experiment 4 shows the results of the model with both the MHLA module and CLS_task added. Comparing Experiment 3 with Experiment 1, it is evident that adding CLS_task led to a slight relative improvement on the commercial flight dataset and a 3.97% improvement on the training flight dataset compared to the baseline model. Comparing Experiment 4 with Experiment 1, it is clear that adding both the MHLA module and CLS_task resulted in more significant improvements, with a 1.55% relative increase on the commercial flight dataset and a 6.1% increase on the training flight dataset compared to the baseline model. Overall, both the MHLA module and CLS_task are beneficial for enhancing the baseline's performance.

#### Verification of Optimization Strategies

The primary objective of this section is to explore the impact on the model's learning ability when employing EBCE, WBCE, BCE, and their combinations, based on the Baseline-MHLA-CLS_task framework. The model's training process was conducted on the transportation flight and training flight control instruction datasets, with tests carried out at the 10th and 20th epochs during the training process. The testing metric was the F1 score, and the experimental results are presented in Table 4. In Experiment 1, the loss function implemented is BCE, while Experiment 2 utilizes WBCE. Experiment 3 adopts EBCE, and Experiment 4 combines both WBCE and EBCE.

**Table 4:** Impact of Different Loss Functions on Model Learning Speed.

Experiment number	Loss function	Commercial flight data		Training flight data	
		10 epoch	20 epoch	10 epoch	20 epoch
1	BCE	0.001	0.562	0.001	0.347
2	WBCE	0.001	0.001	0.001	0.001
3	EBCE	0.404	0.610	0.376	0.492
4	EBCE& WBCE	**0.544**	**0.685**	**0.507**	**0.612**

Significant values are in bold.

From Experiment 1, it is apparent that when only BCE is used, the model learns at a slower pace. The test results at the 10th training epoch indicate that the model had not yet learned useful information at this point. The results from the 20th epoch suggest that the model had begun to learn a small amount of useful information. Experiment 2 indicates that when using only WBCE, the model fails to learn useful information. The primary reason for this is that WBCE is only suitable when the class imbalance ratio of the data is known. Experiment 3 demonstrates that EBCE is effective in accelerating the model's learning speed. It is applicable regardless of whether the ratio of class imbalance is known or unknown. Experiment 4 indicates that the model's learning capabilities can be further improved by utilizing WBCE for tasks with a known class imbalance ratio and EBCE for tasks with an unknown class imbalance ratio.

#### Sensitivity Analysis

The primary objective of this section is to explore the sensitivity of SLKIR to the number of heads in the MHLA mechanism and their corresponding window_size. Experiments were conducted on transportation flight and training flight datasets, with the F1 score as the evaluation metric. The results are presented in Table 5. Notably, Experiment 1 demonstrates the results under a single-head global attention mechanism for comparison.

**Table 5:** Sensitivity Analysis Results Table.

Experiment number	Num_heads&window_size	Commercial flight data	Training flight data
1	1&none	0.820	0.730
2	2&1,2	**0.851**	**0.790**
3	3&1,2,3	0.831	0.723
4	4&1,2,3,4	0.837	0.757

Significant values are in bold.

The experimental results from Table 5 indicate that as the num_heads increases, the overall trend in model performance initially improves and then declines. The model achieves optimal performance when num_heads is set to 2 and window_size to 1 & 2. Overall, SLKIR is quite sensitive to changes in num_heads and their corresponding window_size. Having too many or too few num_heads and window_size can introduce noise, thereby impacting the model's performance.

### Comparative Experiment

In this section, the models Bert-Softmax, Bert-CRF, Lebert-Softmax, Lebert-CRF (sequence labeling methods), and W2NER (a span-based method) were chosen as benchmarks to compare with SLKIR, aiming to explore the accuracy and generalizability of each model. Notably, Lebert-Softmax and Lebert-CRF each have two versions: one based on a non-ATC dictionary and the other on an ATC dictionary. The experimental results are presented in Tables 6 and 7, with Precision (P), Recall (R), and F1 score as the evaluation metrics.

**Table 6:** Test Results of Various Models on the Commercial Flight Dataset.

Experiment number	Model name	P	R	F1
1	Bert-Softmax	0.826	0.801	0.813
2	Bert-CRF	0.822	0.798	0.810
3	Lebert-Sotmax (None ATC dictionary)	0.825	0.796	0.811
4	Lebert-CRF (None ATC dictionary)	0.821	0.801	0.811
5	Lebert-Sotmax (ATC dictionary)	0.823	0.806	0.814
6	Lebert-CRF (ATC dictionary)	0.821	0.803	0.812
7	W2NER	0.834	0.809	0.821
8	SLKIR	**0.854**	**0.849**	**0.851**

Significant values are in bold.

**Table 7:** Test Results of Various Models on the Training Flight Dataset.

Experiment number	Model name	P	R	F1
1	Bert-Softmax	0.709	0.549	0.619
2	Bert-CRF	0.705	0.508	0.591
3	Lebert-Sotmax (None ATC dictionary)	0.767	0.522	0.621
4	Lebert-CRF (None ATC dictionary)	0.696	0.527	0.600
5	Lebert-Sotmax (ATC dictionary)	0.739	0.535	0.621
6	Lebert-CRF (ATC dictionary)	0.719	0.594	0.650
7	W2NER	0.785	0.631	0.700
8	SLKIR	**0.846**	**0.741**	**0.790**

Significant values are in bold.

From Experiments 1 to 4 in Tables 6 and 7, it is evident that models with a CRF layer are less effective than those with a Softmax layer. This is because the boundary words of key information in ATC instructions are locally dependent, and using a CRF layer introduces information from distant words, effectively adding a degree of noise. In contrast, the Softmax layer, by ignoring inter-word dependencies, reduces the introduction of noise and hence performs relatively better. Experiments 3 to 6 in Tables 6 and 7 show that incorporating external ATC instruction dictionary information leads to a slight improvement in model performance, but the cost-effectiveness is low. From Experiments 7 to 8 in Tables 6 and 7, it is clear that SLKIR significantly outperforms the best span-based model, W2NER. In the transportation flight dataset, SLKIR achieves a 3.65% relative increase in the F1 score compared to W2NER, and in the training flight dataset, it achieves a 12.8% relative increase in F1 score.

## Conclusion

To achieve accurate and robust extraction of key information from air traffic control, we propose a small-sample-driven end-to-end deep learning framework. Based on the aforementioned experimental results and analysis, the following conclusions can be drawn.

- (1) Based on the characteristics of ATC instructions, a MHLA mechanism has been developed to capture latent representations related to boundary words of key information. This mechanism allows the model to effectively improve the discrimination accuracy of key information boundary words without the need for external regulatory instruction dictionaries.
- (2) Considering the significant impact of users' external interaction information on the model's understanding of instruction content, we proposed a classification task based on external prompt information to enhance the model's semantic understanding of input instructions. Results indicate that this design significantly improves the model's generalization ability on unseen samples.
- (3) To address two types of class imbalance problems, different loss function optimization strategies have been proposed in this paper. The experimental results show that adopting the appropriate loss function for different imbalance problems can accelerate the learning speed of the model and improve the accuracy of recognition.
- (4) The proposed deep learning framework has achieved superior accuracy and generalizability on two small-sample datasets compared to the best baseline models.
- Although leading results have been achieved, that are still not sufficient for application in real-world scenarios. In the future, we hope to further optimize the model architecture to handle more complex air traffic instruction scenarios, including various non-standard usages and ambiguous instructions.

## Data Availability

Training flight ATC instruction dataset used in this study is available on GitHub, while commercial flight ATC instruction dataset can be obtained upon reasonable request. For requests to access the remaining dataset, please contact the corresponding author at panatc@163.com.

## Code availability

The source code of this paper is available at:https://github.com/PANPANKK/ATC_KIR.
